# Impact of Hospital Strain on Excess Deaths During the COVID-19 Pandemic — United States, July 2020–July 2021

**DOI:** 10.15585/mmwr.mm7046a5

**Published:** 2021-11-19

**Authors:** Geoffrey French, Mary Hulse, Debbie Nguyen, Katharine Sobotka, Kaitlyn Webster, Josh Corman, Brago Aboagye-Nyame, Marc Dion, Moira Johnson, Benjamin Zalinger, Maria Ewing

**Affiliations:** ^1^Cybersecurity & Infrastructure Security Agency, U.S. Department of Homeland Security, Washington, D.C.; ^2^COVID Task Force Support Cybersecurity & Infrastructure Security Agency, U.S. Department of Homeland Security, Washington, D.C.

Surges in COVID-19 cases have stressed hospital systems, negatively affected health care and public health infrastructures, and degraded national critical functions (*1*,*2*). Resource limitations, such as available hospital space, staffing, and supplies led some facilities to adopt crisis standards of care, the most extreme operating condition for hospitals, in which the focus of medical decision-making shifted from achieving the best outcomes for individual patients to addressing the immediate care needs of larger groups of patients (*3*). When hospitals deviated from conventional standards of care, many preventive and elective procedures were suspended, leading to the progression of serious conditions among some persons who would have benefitted from earlier diagnosis and intervention (*4*). During March–May 2020, U.S. emergency department visits declined by 23% for heart attacks, 20% for strokes, and 10% for diabetic emergencies (*5*). The Cybersecurity & Infrastructure Security Agency (CISA) COVID Task Force* examined the relationship between hospital strain and excess deaths during July 4, 2020–July 10, 2021, to assess the impact of COVID-19 surges on hospital system operations and potential effects on other critical infrastructure sectors and national critical functions. The study period included the months during which the highly transmissible SARS-CoV-2 B.1.617.2 (Delta) variant became predominant in the United States.^†^ The negative binomial regression model used to calculate estimated deaths predicted that, if intensive care unit (ICU) bed use nationwide reached 75% capacity an estimated 12,000 additional excess deaths would occur nationally over the next 2 weeks. As hospitals exceed 100% ICU bed capacity, 80,000 excess deaths would be expected in the following 2 weeks. This analysis indicates the importance of controlling case growth and subsequent hospitalizations before severe strain. State, local, tribal, and territorial leaders could evaluate ways to reduce strain on public health and health care infrastructures, including implementing interventions to reduce overall disease prevalence such as vaccination and other prevention strategies, as well as ways to expand or enhance capacity during times of high disease prevalence.

CDC provided data on excess deaths from all causes; data on hospital strain came from the U.S. Department of Health and Human Services (HHS) hospital utilization timeseries dataset.^§^^,^^¶^ Excess deaths were defined as the difference between observed and expected number of deaths during specific periods** (*6*). Hospital strain was measured by ICU bed occupancy.^††^ Negative binomial regression was used to model estimates and calculate the corresponding 95% CI for excess deaths (dependent variable) and hospital strain (independent variable), controlling for state-level differences, during July 4, 2020–July 10, 2021.^§§^ Tests for robustness with inpatient bed occupancy provided similar results across the United States. Statistical analyses were conducted using R software (version 4.0.2; R Foundation). This activity was reviewed by CISA and CDC, and was conducted consistent with applicable federal law, CISA policy, and CDC policy.^¶¶ ^

During July 4, 2020–July 10, 2021, as ICU bed occupancy increased, excess deaths increased 2, 4, and 6 weeks later (p<0.01). The ICU bed occupancy coefficient was 5.69 (z-score = 15.0). Using data from July 1, 2020–July 10, 2021, on excess deaths from all causes and hospital strain, the model predicted that, if ICU bed use nationwide reached 75% capacity an estimated additional 12,000 (95% CI = 8,623–17,294) excess deaths would occur nationally 2 weeks later (Figure), with additional deaths at 4 and 6 weeks (Cybersecurity & Infrastructure Security Agency COVID Task Force, Cybersecurity & Infrastructure Security Agency, unpublished data, 2021). As hospitals exceed 100% ICU bed capacity, 80,000 (95% CI = 53,576–132,765) excess deaths would be expected 2 weeks later with additional deaths at 4 and 6 weeks (Cybersecurity & Infrastructure Security Agency COVID Task Force, Cybersecurity & Infrastructure Security Agency, unpublished data, 2021).***

**FIGURE:**
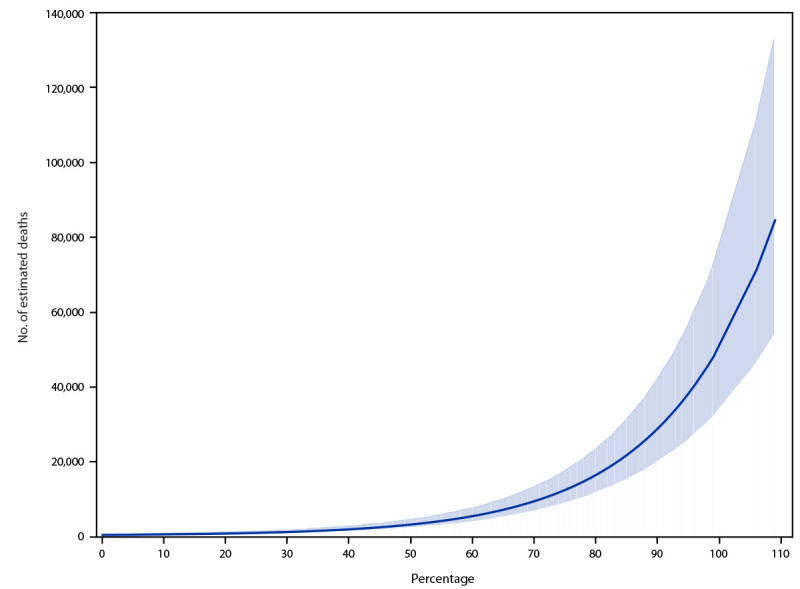
Estimated number of excess deaths* 2 weeks after corresponding percentage of adult intensive care unit bed occupancy — United States, July 2020–July 2021 * Upper and lower boundaries of shaded area indicate 95% CIs.

## Discussion

These findings suggest that ICU bed use is an important indicator, but not the sole contributing factor, of stress to health care and public health sectors, with excess deaths emerging in the weeks after a surge in COVID-19 hospitalizations. The results of this study support a larger body of evidence from previous CISA analyses of the potential consequences of the COVID-19 pandemic on CISA Provide Medical Care National Critical Functions,^†††^ and the cascading effects on the essential critical infrastructure workforce (*7*). Even before COVID-19’s emergence, emergency department crowding, ICU capacity, and ambulance diversion were reported to have adverse outcomes, such as increased medical errors and reduced quality of care (*8*) as well as delays in treatment, medication error, longer patient stays, poorer outcomes, and increased mortality (*9*). During 2020, the impact of these effects, which included potentially avoidable excess deaths, fell more heavily on working-aged adults from marginalized communities who experience poor access to health care outside pandemic conditions (*10*). For example, racial and ethnic subgroups experienced disproportionately higher percentage increases in deaths, with the most pronounced effect among the Hispanic/Latino communities who represent an estimated 21% of the essential critical infrastructure workforce.^§§§^

The nonlinear nature of the curve (Figure) shows how these negative effects increase exponentially as the system becomes more stressed. As of October 25, 2021, per data from the HHS timeseries dataset, capacity in adult ICUs nationwide has exceeded 75% for at least 12 weeks. This means that the United States continues to experience the high and sustained levels of hospital strain that, according to the model’s results, are associated with significant subsequent increases in excess deaths.

The findings in this report are subject to at least three limitations. First, modeling studies are subject to uncertainty, including unforeseen events that could cause deviations from the modeled scenarios. Second, data were incomplete because of the lag in time between when deaths occurred and when death certificates were completed and processed.^¶¶¶^ Finally, although pandemic-driven ICU bed occupancy is not a direct cause of excess deaths, high ICU capacity is a marker of broader issues that can contribute to excess deaths, such as curtailed services, stressed operations, and public reluctance to seek services.

Additional research is warranted to assess the cascading effects of the degraded and disrupted functioning of the health care sector, especially during COVID-19 surges. Studying the nature and extent of these stresses on critical infrastructure and essential critical infrastructure workers**** can help elucidate the consequences of the pandemic and potential ways to address health system vulnerabilities to ensure improved resilience in the future. This analysis indicates the importance of controlling case growth and the subsequent need for hospitalizations before severe strain. State, local, tribal, and territorial leaders could evaluate ways to reduce strain on public health and health care infrastructures, including implementing interventions to reduce overall disease prevalence such as vaccination and other prevention strategies, as well as ways to expand or enhance capacity during times of high disease prevalence.

**Summary**

**What is already known about this topic?**

COVID-19 surges have stressed hospital systems and negatively affected health care and public health infrastructures and national critical functions.

**What is added by this report?**

The conditions of hospital strain during July 2020–July 2021, which included the presence of SARS-CoV-2 B.1.617.2 (Delta) variant, predicted that intensive care unit bed use at 75% capacity is associated with an estimated additional 12,000 excess deaths 2 weeks later. As hospitals exceed 100% ICU bed capacity, 80,000 excess deaths would be expected 2 weeks later.

**What are the implications for public health practice?**

State, local, tribal, and territorial leaders could evaluate ways to reduce strain on public health and health care infrastructures, including implementing interventions to reduce overall disease prevalence such as vaccination and other prevention strategies, and ways to expand or enhance capacity during times of high disease prevalence.

## Footnotes

* The CISA COVID Task Force executes CISA operations in support of the federal COVID-19 response. This includes conducting analyses of COVID-19–related disruptions of national critical functions, including supply chain disruptions and misinformation activity. In addition, the Task Force leverages CISA’s capabilities, services, and relationships to maintain sustained engagement with health care and public health providers.

† https://covid.cdc.gov/covid-data-tracker/#variant-proportions (Accessed November 1, 2021)

§ CDC calculates excess deaths at the state level.

¶ Dataset consists of state-aggregated hospital utilization data in a timeseries format. Sources include the Agency for Health Care Research and Quality, Centers for Medicare & Medicaid, CDC, Food and Drug Administration, and reporting state partners. https://healthdata.gov/Hospital/COVID-19-Reported-Patient-Impact-and-Hospital-Capa/g62h-syeh (Accessed November 1, 2021)

** The dependent variable, excess deaths, is a count variable with significant overdispersion, making the negative binomial regression model the most appropriate one for this analysis. Poisson models were also used to check for robustness, and results were consistent.

†† HHS has studied the relationship between hospital bed use and hospital strain and has identified occupancy >80% as an indicator of a strained condition. This analysis uses a continuous measure of ICU bed occupancy as a proxy for hospital strain, such that greater amounts of ICU bed use indicate larger amounts of hospital strain.

§§ Negative binomial regression model controlled for state differences by including state-level fixed effects. A dummy variable for each state was included in the model to account for observed and unobserved factors at the state level that might drive variation. This is consistent with best statistical practice for understanding variation across administrative units of a country.

¶¶ 45 C.F.R. part 46.102(l)(2), 21 C.F.R. part 56; 42 U.S.C. Sect.241(d); 5 U.S.C.0 Sect.552a; 44 U.S.C. Sect. 3501 et seq.

*** Excess deaths at 4 and 6 weeks correlate with hospital strain, as measured by ICU bed occupancy at a point in time, with numbers decreasing over time at a rate depending on the subsequent duration and severity of hospital strain. Where hospital strain remains high at 4 and 6 weeks after the occurrence of the initial strain, the associated numbers of excess deaths would also remain high.

†††
https://www.cisa.gov/sites/default/files/publications/CISA_Insight_Provide_Medical_Care_Sep2021.pdf

§§§
https://www.epi.org/blog/who-are-essential-workers-a-comprehensive-look-at-their-wages-demographics-and-unionization-rates/

¶¶¶ Death counts were derived from the National Vital Statistics System database that provides the timeliest access to the vital statistics mortality data and might differ slightly from other sources because of differences in completeness. In addition, ICU bed occupancy data are based on the information reported to HHS from participating hospitals and might not be complete.

****
https://www.cisa.gov/publication/guidance-essential-critical-infrastructure-workforce
